# Postmortomics: The Potential of Untargeted Metabolomics to Highlight Markers for Time Since Death

**DOI:** 10.1089/omi.2020.0084

**Published:** 2020-11-04

**Authors:** Bogumila K. Pesko, Stefan Weidt, Mark McLaughlin, Daniel J. Wescott, Hazel Torrance, Karl Burgess, Richard Burchmore

**Affiliations:** ^1^ Glasgow Polyomics, Wolfson Wohl Cancer Research Centre, University of Glasgow, Glasgow, United Kingdom.; ^2^ Veterinary Biosciences, School of Veterinary Medicine, University of Glasgow, Glasgow, United Kingdom.; ^3^ Department of Anthropology, Forensic Anthropology Center at Texas State (FACTS), Texas State University, San Marcos, Texas, USA.; ^4^ Forensic Medicine and Science Department, University of Glasgow, Glasgow, United Kingdom.

**Keywords:** postmortem interval, forensic science, metabolomics, biomarkers, muscle metabolites, amino acids

## Abstract

The success of forensic investigations involving fatalities very often depends on the establishment of the correct timeline of events. Currently used methods for estimating the postmortem interval (PMI) are mostly dependent on the professional and tacit experience of the investigator, and often with poor reliability in the absence of robust biological markers. The aim of this study was to investigate the potential of metabolomic approaches to highlight molecular markers for PMI. Rat and human muscle tissues, collected at various times postmortem, were analyzed using an untargeted metabolomics approach. Levels of certain metabolites (skatole, xanthine, n-acetylneuraminate, 1-methylnicotinamide, choline phosphate, and uracil) as well as most proteinogenic amino acids increased steadily postmortem. Threonine, tyrosine, and lysine show the most predictable evolution over the postmortem period, and may thus have potential for possible PMI markers in the future. This study demonstrates how a biomarker discovery approach can be extended to forensic investigations using untargeted metabolomics.

## Introduction

Estimation of postmortem interval (PMI) plays a central role in the course of many forensic investigations. Unfortunately, it is also one of the most challenging tasks in crime investigation. Knowing when death occurred may help clarify the circumstances of a death and to assess the alibis of potential suspects in a suspicious death (Kaliszan et al., 2009). However, current methods to determine PMI, including temperature measurement (algor mortis), assessment of rigor mortis and livor mortis (lividity), and morphological changes in the body (Swift, 2006), are often inaccurate and depend greatly on the tacit professional experience of the forensic investigator (Aydin et al., 2010).

Various other methods have been proposed over the years (Kang et al., 2003; Lange et al., 1994; Rognum et al., 1991; Sampaio-Silva et al., 2013; Shirley and Wood, 2013; Usumoto et al., 2010), but with limited success due to the complexity of the decomposition process. Margins of error in field forensic assessments of PMI can exceed 40% (Kaliszan et al., 2009), even when conventional methods are combined. There is a clear need for a reliable, quantitative, and objective method for PMI estimation.

Global or “omics” systems science approaches, such as proteomics and metabolomics, have led to the identification of biomarkers that have diagnostic or prognostic utility to forecast health and disease states (Blasco et al., 2014; Fedele et al., 2013; González-Domínguez et al., 2014; LeWitt et al., 2013; Manna et al., 2015; Pena et al., 2015). Untargeted metabolomics can report on the relative abundances of a broad range of small molecules (such as amino acids, oligopeptides, sugars, bile acids, and fatty acids) in any biological system of interest (Clarke and Haselden, 2008).

Various metabolites have previously been investigated for use in PMI estimation. Harada et al. (1984) investigated the changes in levels of specific metabolites (lactate, alanine, pyruvate, and glucose) in animal organs and tissues (liver, kidney, spleen, brain, heart, and dorsal muscle) and found a correlation between PMI and levels of lactate and pyruvate in the heart and muscle tissue. Sparks et al. (1986) presented a linear relationship between 3-methoxytyramine (3-MT) and PMI in the dorsal putamen. The authors also concluded that this relationship varies with the cause of death. In cases where the cause of death was attributed to a heart disease, 3-MT was negatively correlated with PMI, while being positively correlated in cases with other causes of death.

Four other studies reported metabolites that evolve over time in postmortem sheep brain (Ith et al., 2002; Ith et al., 2011; Musshoff et al., 2011; Scheurer et al., 2005). Free trimethylammonium (fTMA), propionate, and butyrate were found in all three investigations, among other compounds emerging postmortem. Ith et al. (2002) also included human brain in the study, and found similarities between the decomposition of human and sheep brain. Banaschak et al. (2005) investigated the postmortem changes in pig brain, and also identified fTMA in their study, as well as creatinine, lactate, acetate, alanine, and succinate, which all emerged in postmortem tissue.

More recently, Mao et al. (2013) showed a correlation between PMI and the breakdown products of adenosine triphosphate in rat brain, kidney, and spleen. Donaldson et al. (2013) observed that the concentrations of hypoxanthine, ammonia, formic acid, and nicotinamide adenine dinucleotide (NADH) increase in rat, pig, and human blood postmortem. Kang et al. were one of the first groups to utilize a global mass spectrometry-based metabolomics approach (as opposed to metabolite assays) in PMI estimation. Metabolites extracted from rat livers over the first 48 h postmortem were analyzed by ultra performance liquid chromatography/quadrupole-time of flight mass spectrometry (UPLC/Q-TOF MS). Statistical analysis of the data yielded 15 potential biomarker candidates, including various polysaccharides, steroids, nucleosides, and amino acids (Kang et al., 2012).

We used an untargeted comparative metabolomics approach to identify putative molecular markers that predictably evolve over time after death. The instrumentation most commonly employed for metabolomics includes liquid chromatography-mass spectrometry (LC-MS) and gas chromatography-mass spectrometry. These techniques allow identification and relative quantitation of low molecular weight molecules (up to ∼1000 Da). Here, we report the development of a mass spectrometry-based metabolomics approach to identify PMI biomarkers in a rodent model and the application of this approach to human cadavers with known PMI.

## Materials and Methods

### Research ethics

This study involved both human and animal postmortem samples. The six cadavers used in this research project were donated to the Forensic Anthropology Center at Texas State (FACTS) University, San Marcos, Texas. The donations were made voluntarily in life by the individuals themselves or by next of kin after death, in accordance with the Texas Anatomical Gift Program. All legal documents permitting the release of the remains to FACTS and informed consent were obtained for each subject.

Work with human remains was performed with the approval of Texas State University. FACTS receives whole-body donations for scientific research under the Texas revised Uniform Anatomical Gift Act (Texas Health and Safety Title 8, Chapter 691 and 692). Body donations are exclusively acquired by Texas State University through the expressed and documented willing of the donors and/or their legal next of kin. Body donations are made directly to FACTS, and donors and/or their legal next of kin are aware that donations are used for taphonomic studies. Upon receipt of a body donation, a number is assigned to the donation, and the name and personal information are not accessible to the researcher. The body donation program complies with all legal and ethical standards associated with the use of human remains for scientific research.

Human tissue samples were collected according to guidelines approved by FACTS, which complied with all legal and ethical standards associated with the use of human remains for scientific research, as detailed above. The present work was conducted within the oversight of all host organizations. The local research ethics committee chair at the University of Glasgow where molecular analyses were performed has advised the authors and waived the ethics approval in Glasgow, in light of ethics approval at the FACTS, the U.S. collaborating center.

All human subjects admitted to the FACTS during a 2-week period were included in the study, provided their lower limbs were intact and suitable for sampling. Basic anonymous information on the individuals, including the cause and time of death, was recorded, as shown in Table 1. All applicable international, national, and/or institutional guidelines for the care and use of animals were followed. All animal postmortem samples were obtained after animals were euthanized strictly adhering to Schedule 1 of the UK Animals (Scientific Procedures) Act 1986.

**Table 1.** Description of the Human Cadavers Included in the Study

Subject ID	Gender	Age	Cause of death	Sampling period (days)	PMI (days)
1^*^	Male	60	Hemorrhagic stroke	4	7, 8, 9, 10
2^*^	Male	62	Pulmonary embolism	4	11, 12, 13, 14
3^*^	Female	69	Suicide—GSW (chest)	5	11, 12, 13, 14, 15
4	Male	59	Acute respiratory distress syndrome	3	3, 4, 5
5^*^	Male	69	Metastatic nonsmall cell lung cancer	5	12, 13, 14, 15, 16
6	Male	60	Cardiovascular disease	2	18, 19

Cadavers stored in refrigerated conditions are marked with an asterisk.

PMI at the time of sampling.

GSW, gun-shot wound; PMI, postmortem interval.

### Sample collection

#### Rat tissue

Initially, rat cadavers were used as an animal model to test the methodology. Eight male adult Wistar rats (283–309 g, average weight 296.5 g) were euthanized through carbon dioxide asphyxiation, per Schedule 1 of the UK Animals (Scientific Procedures) Act 1986, as noted above.

Two of the rats were immediately dissected, and the biceps femoris muscle tissue was collected from both left and right sides of the body, giving four replicate tissue samples from two animals. The six remaining rat cadavers were stored in a covered plastic box at 20°C (±2°C), and were dissected as described above at 24 h intervals postmortem over three consecutive days (two rats per day, four samples per day). The collected tissue was immediately weighed, cut into 0.5 g portions, placed in separate cryogenic vials (Nunc CryoTubes, 1.8 mL; Sigma-Aldrich, Dorset, UK), and snap frozen in liquid nitrogen. Samples were then stored at −80°C until analysis.

#### Human tissue

Samples were collected from human cadavers at a similar time each day of sampling. The days on which samples were collected postmortem from each cadaver are listed in Table 1. Four of the six cadavers (subjects 1, 2, 3, and 5) were refrigerated (in body bags) throughout the period of sampling at 7.2°C (45F). This temperature is routinely used to refrigerate cadavers at FACTS. Shortly before sampling, the bodies were removed from the fridge, right leg of the cadaver was lifted up, and a single incision was made at the back of the leg, along the biceps femoris muscle line. The underlying muscle tissue (biceps femoris) was exposed, and a piece of the muscle was cut out using a scalpel. The collected tissue was then weighed into six 1 g (approximately) portions and put into separate screw top vials (microtubes 2 mL; PP—Sarstedt, Leicester, UK).

The incision was covered with surgical tape, and the cadavers were returned to the refrigerator immediately after sampling. Subsequent sampling was performed from the same incision to avoid further invasive procedures. The bodies were returned to the refrigerator immediately after sampling. The collected samples were kept on dry ice for ∼1.5 h until they were stored at −80°C.

The other two individuals (subjects 4 and 6) were placed outdoors, and facing down for easy access to the sampling site, and preserved in a secure exposed environment. During the 5-day period when these two cadavers were sampled, the average temperature was 23.3/17.8°C (day/night) and the average relative humidity was 61.7/85.9% (day/night). An incision was made to expose the muscle, and a piece of biceps femoris was collected. The tissue was immediately wrapped in a piece of aluminum foil and placed in a sealed plastic bag to be transported to the laboratory. The incision site was covered up with tape to prevent the development of insect activity in the wound.

At the laboratory, the tissue was cut up and weighed into six portions of ∼1 g. They were then placed in vials and kept on dry ice for ∼1.5 h, before being stored in a −80°C freezer until shipment to the United Kingdom where they were also stored at −80°C. The muscle tissue was processed and analyzed at the University of Glasgow.

### Sample preparation

While this study did not conduct formal statistical hypothesis testing due to limited sample size, it offers important and new insights on PMI research. Here, we describe, additionally, how data were organized and reported. Four separate pieces of rat tissue (two rats, right and left leg) and three separate pieces of human tissue were used for each time point.

Tissue samples (0.5 g) were homogenized in 5 mL of chloroform/methanol/water solution 20:60:20 (v/v/v) (t'Kindt et al., 2010) using a handheld homogenizer (Cole-Palmer) and shaken on ice for 1 h using a rocking table (150 rpm; Lab-Shaker LS-X, Kuhner, Switzerland). After extraction, samples were centrifuged at 14,000 *g* for 30 min. One milliliter of the supernatant was transferred to an Eppendorf tube and centrifuged again at 16,000 *g* for 10 min. Samples were then transferred to fresh Eppendorf tubes and stored at −80°C until analysis.

### Sample analysis

Ten microliters of each sample was used for analysis using hydrophilic interaction LC-MS (UltiMate 3000 RSLC; Thermo Scientific, San Jose, CA) with a 150 × 4.6 mm ZIC-pHILIC analytical column (Merck SeQuant, Umea, Sweden) running at 300 μL/min, coupled to an Exactive (Thermo Scientific) for MS detection. Buffers consisted of A: 20 mM ammonium carbonate (Sigma Aldrich) in H_2_O (LC-MS Grade; Rathburn, Walkerburn) and B: acetonitrile (LC-MS Grade; Rathburn). The gradient ran from 80% B to 20% B in 15 min, followed by a wash at 5% B for 3 min, and equilibration at initial conditions for 5 min. Column conditions were maintained in a column oven at a fixed temperature of 25°C.

The Exactive (Thermo Scientific, Waltham, MA) was used to detect ions using a HESI-II interface with a source temperature of 150°C and a capillary temperature of 270°C. Sheath gas flow rate was 40, auxiliary gas flow rate was 5, and sweep gas flow rate was 1. Each sample was injected using alternating detection in positive ion and negative ion using polarity switching mode; the spray voltage was 4.5 and 3.5 kV, respectively. Full scan MS detection was acquired for the range 70 to 1400 m/z at 50,000 (at 400 m/z) resolution for each polarity.

For better detection and identification of different groups of compounds, the samples were also analyzed using a ZIC-HILIC analytical column (150 × 4.6 mm; Merck SeQuant) coupled to the same LC-MS system. Method parameters were the same as above with the exception of the buffers used—A: 0.1% formic acid and B: 0.08% formic acid, both in acetonitrile.

Raw mass spectrometry data were processed using the University of Glasgow Polyomics pipeline, consisting of XCMS (Smith et al., 2006) (for peak picking), MzMatch (Scheltema et al., 2011) (for filtering and grouping), and IDEOM (Creek et al., 2012) (for further filtering, postprocessing, and identification). Core metabolite identifications were validated against a panel of unambiguous standards by mass and retention times. Additional putative identifications were assigned by accurate mass along with a retention time prediction algorithm (Creek et al., 2011). Mean and standard deviation of the mean were generated for all groups of picked peaks.

### PMI calculations

PMI is most often reported using the amount of time, which has passed since the moment of death. However, the temperature at which the cadaver decomposes has a significant effect on the rate of decomposition. Therefore, the PMI calculation is sometimes corrected to include the temperature measurement. For example, Vass et al. (2002) reported PMI values in cumulative degree hours (CDH). To allow for comparison of results presented in this article with the existing literature, the PMI of subjects included in this study is also presented in CDH (Table 2).

**Table 2.** Sampling Times for Human Subjects Included in this Study as Postmortem Interval (in Days) and Cumulative Degree Hour

	Unit	Time point 1	Time point 2	Time point 3	Time point 4	Time point 5
Subject 1	PMI	7	8	9	10	X
	CDH	81.6	96.0	110.4	124.8	X
Subject 2	PMI	11	12	13	14	X
	CDH	88.0	102.4	116.8	131.2	X
Subject 3	PMI	11	12	13	14	15
	CDH	107.2	121.6	136.0	150.4	164.8
Subject 4	PMI	3	4	5	X	X
	CDH	137.4	178.9	222.4	X	X
Subject 5	PMI	12	13	14	15	16
	CDH	108.8	123.2	137.6	152.0	166.4
Subject 6	PMI	18	19	X	X	X
	CDH	144.0	186.0	X	X	X

CDH was calculated according to Vass et al., 2002.

X indicates that no data were collected for this time point.

CDH, cumulative degree hours.

Calculations were based on the assumption that the human cadavers were stored at 4°C at the medical examiner's office in between the discovery and receipt at the FACTS. The calculations of CDH for the storage period at 4°C were summed with the storage at FACTS (at 7.2°C) to reflect the change in temperature. For cadavers stored outside, actual environmental temperature measurements were used for CDH calculations.

## Results

Untargeted metabolomics approach was used to identify possible biomarkers of PMI. Rat and human skeletal muscle samples were collected at varying times postmortem and analyzed as a time course. This approach allowed the detection and putative identification of 1753 (pHILIC) and 1080 (HILIC) metabolites in rat tissue and 1393 (pHILIC) and 1464 (HILIC) for the human subjects. These numbers reflect the peaks that could be matched within 3 ppm of calculated metabolite masses, and are therefore, class II identifications according to the Metabolite Standards Initiative (Sumner et al., 2014).

**FIG. 1.**
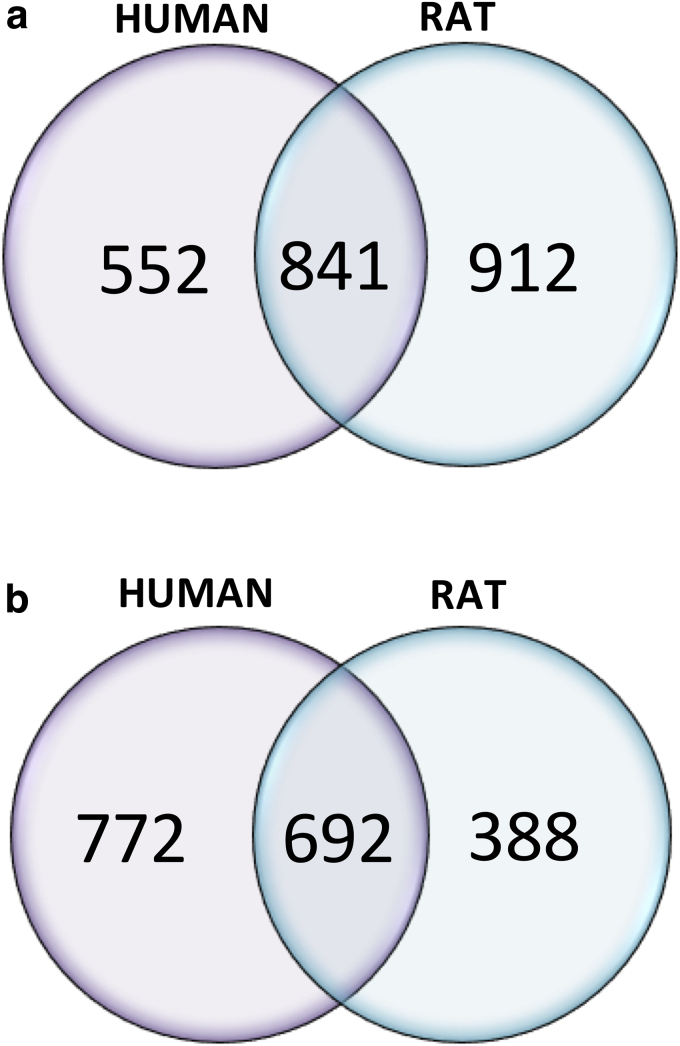
Venn diagrams showing the number of common and uncommon metabolites detected in rat and human tissues. Graph **(a)** shows metabolites separated using pHILIC and **(b)** shows metabolites separated using HILIC.

The majority of the detected molecules were peptides. The other groups of detected metabolites involved diverse pathways, including amino acid, carbohydrate, nucleotide, and lipid metabolism, as well as molecules with no confirmed metabolic role. Figure 1 shows the number of common metabolites between the rat and the human tissue for each chromatographic method.

### Rat tissue

#### Amino acids

**FIG. 2.**
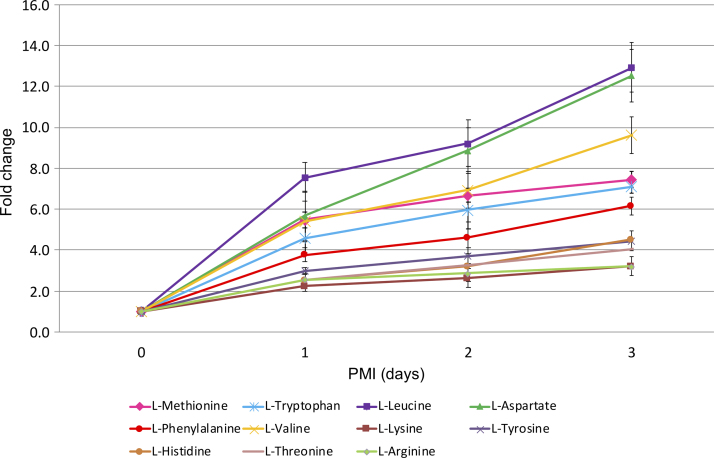
Fold change in signal for 11 amino acids in rat tissue with increasing PMI. Signal at each time point was normalized to time 0. Error bars indicate standard deviation between four samples. Two rats were analyzed per time point, from each hind limb, giving four replicate samples at each time point. PMI, postmortem interval.

Nineteen of 20 of the proteinogenic amino acids were identified (with the exception of isoleucine). Sixteen of them showed steadily increasing patterns over PMI. The remaining three (glycine, alanine, and glutamine) showed less change over the investigated time period. Figure 2 shows the changes observed in 11 of those amino acids, which were identified with high confidence.

Among all of the detected amino acids, cysteine showed the highest rate of increase, ∼22-fold by the third-day postmortem. However, it is not shown in Figure 2 as a potential biomarker candidate as the calculated standard deviation at one of the time points was very high (20.03 ± 10.21). Leucine and aspartate increased 13-fold in that time, followed by valine with a 10-fold increase. Most of the other amino acids increased between four- and eightfold with proline increasing at the slowest rate of twofold change over the same time period and arginine and lysine threefold.

In addition, since two rat cadavers were sampled at each time point, their metabolite levels were compared with each other. The relative levels of the metabolites differed; however, the rates of increase of four amino acids (histidine, lysine, proline, and asparagine) showed similarities between rats sampled at the same time point.

#### Decomposition products

**FIG. 3.**
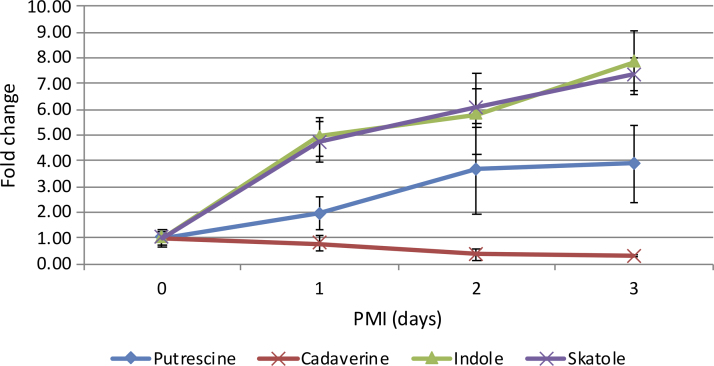
Patterns of change in identified decomposition products with increasing PMI in rat tissue shown as fold change with respect to time of death, with error bars indicating the standard deviation (*n* = 4).

Biogenic amines, such as cadaverine, putrescine, indole, and skatole, are well-known decomposition products (Vass, 2001). They are formed by bacterial breakdown of amino acids after death but are also present in living tissue (Wishart et al., 2013). Cadaverine is a product of lysine metabolism, putrescine of arginine and proline metabolism, and both indole and skatole are produced from tryptophan. All four of these compounds were detected. Putrescine, skatole, and indole increased in abundance over the 3 day time course by ∼4-, 7-, and 8-fold, respectively. Unexpectedly, cadaverine decreased by ∼3-fold over the 3 day time course (Fig. 3).

#### Other metabolites

**FIG. 4.**
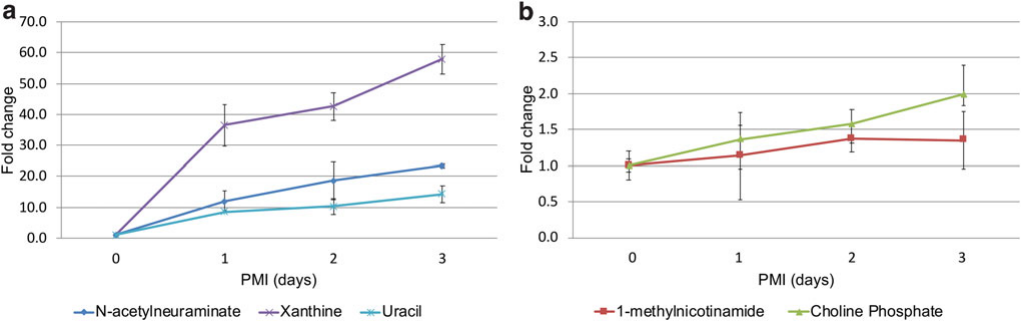
Metabolites that increased in rat tissue with increasing PMI shown as fold change with respect to time of death, with error bars indicating the standard deviation (*n* = 4). The metabolites above, with the exception of 1-methylnicotinamide, were identified by matching to a reference standard. Panels **a** and **b** present different scales.

Five other compounds of interest were selected as potential markers of the PMI based on their steadily increasing change over time as well as on the confidence in the compound identification: xanthine (from purine metabolism), n-acetylneuraminate (a derivative of neuraminic acid), uracil (from pyrimidine metabolism), choline phosphate (from glycerophospholipid metabolism), and 1-methylnicotinamide (a metabolite of nicotinamide) (Wishart et al., 2013). Xanthine displayed the most significant increase of ∼58-fold over 3 days postmortem. N-acetylneuraminate and uracil showed a slower, but still significant, rate of increase, ∼24- and 15-fold, respectively (Fig. 4a). Choline phosphate also increased consistently over the investigated period but by a smaller factor of twofold (Fig. 4b).

### Human tissue

Human tissue was prepared and analyzed in the same way as rat tissue, with the exception of data processing. In the case of rat tissue, rats dissected at the same time point were treated as biological replicates, and data for all cadavers were analyzed collectively. Whereas due to the high variability between individual human cadavers (due to cause of death, age, sex, diet, environmental exposure since death, *etc.*), the data collected for each cadaver were processed as an individual time course to demonstrate changes in the metabolome in comparison with baseline.

#### Amino acids

**FIG. 5.**
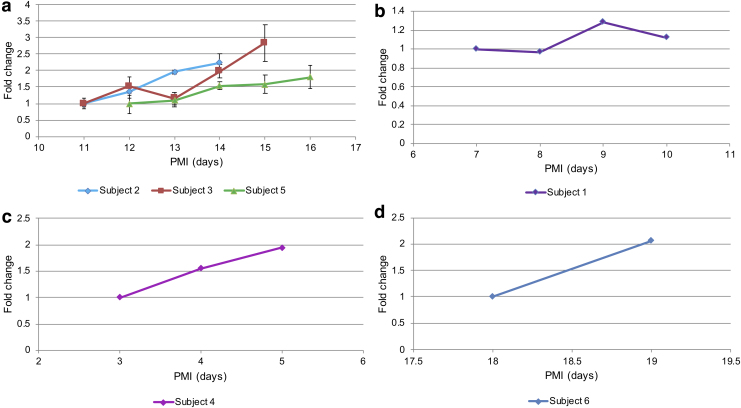
Changes in leucine, as an exemplar marker, over PMI for several human cadavers. Leucine levels are shown as fold change with respect to time of death, normalized to the level of leucine in the earliest sample available for each subject: **(a)** subjects 2, 3, and 5 (similar PMI); **(b)** subject 1; **(c)** subject 4; and **(d)** subject 6. A steady increase in leucine level can be seen for all of the subjects, with exception of subject 1. The analysis included three technical replicates of each sample.

As was the case for rat tissue, generally increasing patterns for most of the amino acids were observed for all of the tested human cadavers, with the exception of subject 1. Figure 5 shows an example of how the levels of one of the amino acids of interest (leucine) changed over time in each of the subjects. Similar trends were observed for nearly all of the proteinogenic amino acids with the exception of glutamine and alanine. Not every metabolite behaved the same in each test subject, but leucine, tryptophan, tyrosine, threonine, lysine, phenylalanine, and aspartate showed steady increases in all. The quantitative changes in amino acid levels were more variable between human individuals than the rat subjects (Fig. 2).

**FIG. 6.**
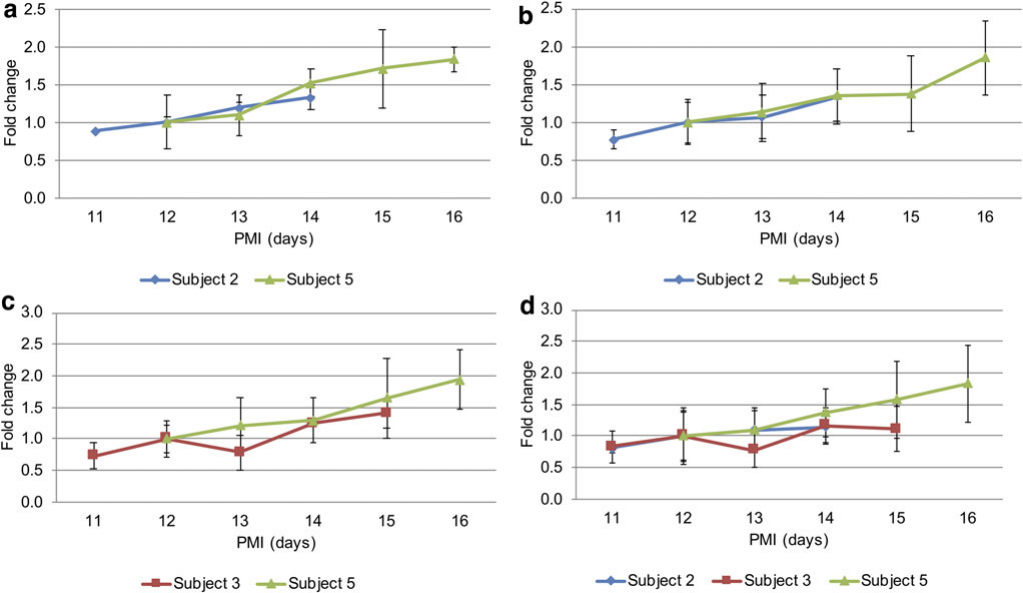
Selected amino acids showing a similar trend of increase over time for different human subjects at the same PMI: **(a)** glutamate, **(b)** histidine (two male subjects), **(c)** lysine (male and a female), and **(d)** serine (all three subjects). Signal was normalized to PMI 12 (first common time point for all subjects). Error bars indicate standard deviation between three technical replicates for each sample.

Although similar trends were observed in all subjects, it was difficult to draw conclusions based on this particular set of subjects due to differences in storage conditions of the cadavers as well as their different PMIs. The instrumental response of amino acids for subjects 2, 3, and 5 (who were of similar age and PMI) was normalized to PMI 12 in each subject (the first common time point for all of the three subjects). These are shown in Figure 6. Four amino acids (glutamate, histidine, lysine, and serine) show similar rates of increase for the two male subjects, while lysine and tyrosine show an overlap for subjects 3 and 5—female and male, respectively. Cysteine and serine show an overlap for all three subjects. This finding is in agreement with data collected from rat cadavers, especially for histidine and lysine, which showed similar rates of increase in individual rat subjects.

Tyrosine, threonine, and lysine, among the amino acids, showed the most consistent increases over time between different individuals. In addition, as mentioned above, they showed similar rates of change for subjects of similar age and PMI, suggesting that they are promising candidates for PMI biomarkers.

#### Other metabolites

The known decomposition products cadaverine, putrescine, and indole, which showed promising changes in rat tissue, did not show similar changes in human tissue. In some cases, no changes were detected. However, another decomposition product (skatole) was consistently identified in five of the six tested subjects and showed increasing patterns for four of the five subjects (with the exception of subject 1).

As in the case of rat tissue, five metabolites (xanthine, choline phosphate, uracil, n-acetylneuraminate, and 1-methylnicotinamide) showed steadily increasing patterns in each of the subjects. Generally increasing patterns were observed for all of these metabolites in each of the cadavers, with the exception of subject 4. However, in this case tissue collection was only possible at three time points, and the cadaver was stored outdoors. N-acetylneuraminate showed the highest rate of increase over time for five of the six subjects (with the exception of subject 3).

**FIG. 7.**
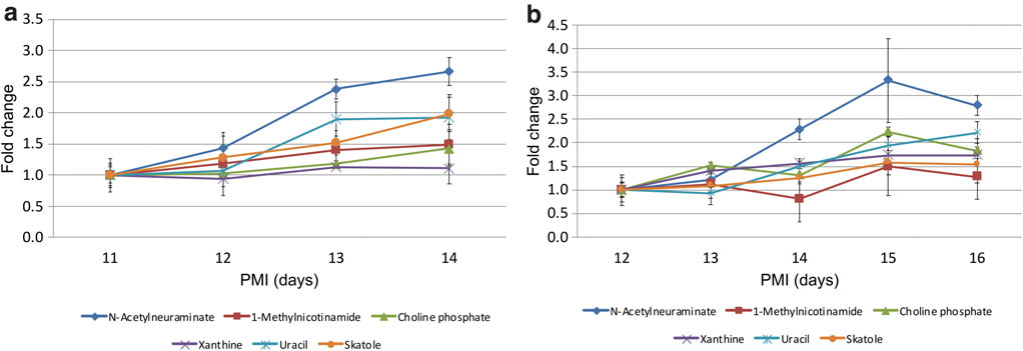
Graphs showing examples of PMI changes in human cadavers of levels of metabolites of interest (n-acetylneuraminate, 1-methylnicotinamide, choline phosphate, xanthine, uracil, and skatole), shown as fold change with respect to time of death. Data shown for two of the human cadavers: **(a)** subject 2 and **(b)** subject 5, normalized to signal in the earliest sample available for each subject. Error bars indicate standard deviation between three technical replicates for each sample.

Figure 7 shows examples of the changes observed in these metabolites for subjects 2 and 5. In addition, since the PMI covered by the study varied for each cadaver from as little as 3 days postmortem to 19 days after death, a general idea could be obtained of how these metabolites changed throughout this period. They show an increasing trend in all cases but subject 4 (which was analyzed to PMI 5), suggesting that these metabolites increase later in the postmortem period (between 7 and 19 days postmortem).

Metabolites of interest were selected based primarily on the predictable and steady rate of change in their levels with increasing PMI as well as the confidence in the metabolite identification. Furthermore, selected metabolites have shown similar trends between the tested human subjects and the rat tissue, and reinforced the confidence in the selected compounds. Metabolites of interest include skatole (3-methylindole), xanthine, n-acetylneuraminate, 1-methylnicotinamide, choline phosphate, uracil, and a number of amino acids (Table 3) with tyrosine, threonine, and lysine being the most promising candidates.

**Table 3.** All Identified Metabolites of Interest

Metabolite name	Formula	KEGG ID	Detected mass	RT (min)	Metabolite ID code
L-Arginine	C_6_H_14_N_4_O_2_	C00062	174.1116	25.79	HRMS^1^_a_, R_ta_
L-Histidine	C_6_H_9_N_3_O_2_	C00135	155.0696	14.59	HRMS^1^_a_, R_ta_
L-Leucine	C_6_H_13_NO_2_	C00123	131.0946	10.12	HRMS^1^_a_, R_ta_
L-Lysine	C_6_H_14_N_2_O_2_	C00047	146.1055	24.39	HRMS^1^_a_, R_ta_
L-Methionine	C_5_H_11_NO_2_S	C00073	149.0511	10.83	HRMS^1^_a_, R_ta_
L-Phenylalanine	C_9_H_11_NO_2_	C00079	165.079	9.54	HRMS^1^_a_, R_ta_
L-Proline	C_5_H_9_NO_2_	C00148	115.0633	11.96	HRMS^1^_a_, R_ta_
L-Threonine	C_4_H_9_NO_3_	C00188	119.0583	13.52	HRMS^1^_a_, R_ta_
L-Tryptophan	C_11_H_12_N_2_O_2_	C00078	204.09	10.94	HRMS^1^_a_, R_ta_
L-Tyrosine	C_9_H_11_NO_3_	C00082	181.0739	12.2	HRMS^1^_a_, R_ta_
L-Valine	C_5_H_11_NO_2_	C00183	117.079	11.68	HRMS^1^_a_, R_ta_
Putrescine	C_4_H_12_N_2_	C00134	88.1001	21.97	HRMS^1^_a_, R_ta_
Xanthine	C_5_H_4_N_4_O_2_	C00385	152.0334	9.03	HRMS^1^_a_, R_ta_
Choline phosphate	C_5_H_14_NO_4_P	C00588	183.0662	17.52	HRMS^1^_a_, R_ta_
Uracil	C_4_H_4_N_2_O_2_	C00106	112.0273	7.985	HRMS^1^_a_, R_ta_
L-Asparagine	C_4_H_8_N_2_O_3_	C00152	132.0535	14.48	HRMS^1^_a_
L-Aspartate	C_4_H_7_NO_4_	C00049	133.0375	14.0	HRMS^1^_a_
L-Cysteine	C_3_H_7_NO_2_S	C00097	121.0198	13.09	HRMS^1^_lib_
L-Glutamate	C_5_H_9_NO_4_	C00025	147.0532	9.59	HRMS^1^_a_
L-Serine	C_3_H_7_NO_3_	C00065	105.0426	14.97	HRMS^1^_a_
Cadaverine	C_5_H_14_N_2_	C01672	102.1157	22.08	HRMS^1^_lib_
Indole	C_8_H_7_N	C00463	117.0579	10.59	HRMS^1^_lib_
Skatole	C_9_H_9_N	C08313	131.0735	10.94	HRMS^1^_lib_
N-acetylneuraminate	C_11_H_19_NO_9_	C00270	309.1061	12.23	HRMS^1^_a_
1-Methylnicotinamide	C_7_H_8_N_2_O	C02918	136.0637	24.5	HRMS^1^_lib_

KEGG ID—number corresponding to a given metabolite in Kyoto Encyclopedia of Genes and Genomes (Kanehisa, 1995), RT—retention time, Metabolite ID code—suggested coding system for reporting confidence of metabolite identification (Sumner et al., 2014), R_ta_ signifies retention time matched identifications (high confidence); the remaining are putatively identified.

## Discussion

An untargeted metabolomics approach allowed the identification of a number of metabolites that show potential as PMI biomarkers. Two chromatographic methods were used: pHILIC and HILIC. The numbers of common metabolites for both rat and human detected by each chromatographic method were presented (Fig. 1), illustrating the enhanced metabolomic coverage that can be achieved by combining two chromatographic separations. A comparable number of metabolites were detected overall from each tissue, but there is a noticeable difference in the number of metabolites detected by the HILIC method in the rat model. This might reflect differences in the kinetics of postmortem degradation in the much smaller rodent cadavers, but more data would be needed to draw any definite conclusions.

Testing carried out using rat cadavers resulted in a number of putative markers, mainly proteinogenic amino acids. Nearly all of the identified amino acids showed a steady rate of increase during the first 72 h after death. Protein degradation may explain many of the changes observed in this study. Since there is no more protein synthesis occurring in dead tissue, the pool of free amino acids does not deplete. The loss of homeostasis that accompanies death will rapidly compromise membrane functions that are actively maintained, leading to breakdown of cellular and subcellular structure. The many proteases that are sequestered in living tissue will gain unregulated access to protein substrates, and the resulting proteolysis will break down proteins into peptides and, ultimately, amino acids.

Degradation of macromolecules is to be anticipated during the extending PMI, and we see many changes (both increases and decreases) in putative markers. Identification of these molecules was beyond the scope of the current work, but suggests the possibility that a panel of chemically diverse markers might be developed to report on PMI.

Well-known bacteria-induced decomposition products, cadaverine, putrescine, skatole, and indole, also showed promising patterns of change in the rat. The latter three compounds increased over time, but cadaverine, surprisingly, decreased. It is possible that the bacterial action was not sufficiently advanced to yield significant amounts. In addition, cadaverine is a metabolite of lysine, which showed one of the slowest rates of increase among the amino acids observed.

Five other metabolites were also identified as possible murine biomarkers: xanthine (a purine base), uracil (a pyrimidine found in RNA), choline phosphate (the precursor of choline in glycine, serine, and threonine pathways), n-acetylneuraminate (otherwise known as sialic acid, a derivative of the amino sugar neuraminic acid), and 1-methylnicotinamide (a metabolite of nicotinamide) (Wishart et al., 2013). All of these compounds increased with PMI.

These findings were also tested using human tissue at various stages of decomposition, where subject selection, cause of death, and ante- and postmortem environment could not be controlled but where the PMI was significantly longer than in our rat study (up to19 days after death). Nonetheless, the data collected using human subjects broadly agreed with the data obtained from rats. The same compounds were identified in most of the cadavers and showed similar trends. The only exceptions were likely microbial decomposition products (cadaverine, putrescine, and indole), which showed less apparent changes in human tissue, perhaps because microbial colonization of the larger human skeletal muscles is delayed compared with rats. On the contrary, skatole showed an increasing trend in human tissue.

In addition, certain amino acids (especially histidine and lysine) showed comparable rates of increase between individual rat and human subjects. The quantitative changes in the levels of amino acids in human tissue were more variable compared with rats, likely because the rat study was more controlled in subject and environment. In addition, the human cadavers were sampled at a longer PMI and were therefore likely in a more advanced stage of decay. The same muscle was sampled repeatedly from each subject, allowing exposure to the environment between the times of sampling.

Amino acids have previously been suggested as possible markers for PMI estimation. In agreement with this study, increasing levels of amino acids were reported in various postmortem specimens, including vitreous humor (Girela et al., 2008; Patrick and Logan, 1988), cerebrospinal fluid (Girela et al., 2008; Kärkelä and Scheinin, 1992), blood (Donaldson and Lamont, 2014; Sato et al., 2015), and brain (Perry et al., 1981). Perry et al. (1981) observed postmortem increases in levels of various amino acids in rat and human brain samples up to 48 h after death. These investigators described a rapid increase in most of the proteinogenic amino acids within 24–48 h postmortem, with the exception of glutamate. This is consistent with our observations, where glutamate was not reliably detected in our rat study, performed over a comparable time period, and showed a modest increase in human cadavers.

Vass et al. (2002) investigated various compounds in different postmortem organs and tissues, including muscle tissue. Similar to our findings in human tissue, this group found the most commonly known decomposition products (cadaverine and putrescine) to be unsuitable for time since death determination due to their variability. The authors described various amino acids as good biomarker candidates. However, according to their findings, muscle tissue can only be used to determine PMI >300 CDH. This is contrary to our findings, as we found amino acids to be useful biomarkers at earlier PMI. To put this into context, in this study subject 2 was sampled between 88 and 131.2 CDH (11–14 days postmortem) and subject 3 107.2 and 164.8 CDH (11–15 days postmortem).

In addition, we found lysine to be one of the most promising biomarker candidates, while Vass et al. (2002) did not find lysine to be a consistent indicator of PMI in any of the tissue types analyzed.

To our knowledge, xanthine, uracil, choline phosphate, n-acetylneuraminate, and 1-methylnicotinamide have not previously been suggested as possible PMI markers. Lendoiro et al. (2012) presented an LC-MS/MS method of PMI estimation based on the measurement of hypoxanthine levels in vitreous humor. The method was also validated to quantify levels of xanthine, however, not as a potential marker. Another study by Kovács et al. (2005) showed increasing levels of both xanthine and uracil during the first 24 h postmortem in rat and human brains. These findings are in agreement with the data presented in this article. However, PMI determination was not the objective of their investigation.

## Conclusions

In this study, we aimed to test the potential for metabolomics to reveal markers for PMI, and we did not have sufficient resource to assess the large numbers of subjects that would be required to generate markers that might be employed with confidence in forensic investigations. Nevertheless, large clinical metabolomic studies, involving hundreds of subjects, can report on a large range of metabolites and reveal markers for complex pathologies (Rattray et al., 2019). Such approaches could readily be applied to postmortem samples.

Muscle tissue has rarely been the focus of PMI research; however, it has veritable potential as a forensic specimen. Superficial muscle tissue is very easy to collect and less emotive for the next of kin. In addition, it is more stable postmortem than other organs or body fluids. As demonstrated in this study, it can be easily applied in PMI investigations. Various compounds show a steady increase over time, with threonine, tyrosine, and lysine showing the most potential as biomarkers.

However, more work is required to validate these results. The next step would be to apply targeted analysis in a larger cohort of subjects to evaluate the selected metabolites. Quantification of the concentrations of these compounds would allow statistical analysis of the data and possibly the development of a mathematical model for the estimation of PMI using muscle tissue. Importantly, instrumentation and methods for the quantification of amino acids in biological samples are well developed and are ubiquitous in clinical biochemistry laboratories.

Taken together, this study demonstrates how a biomarker discovery approach can be extended to forensic investigations using untargeted metabolomics.

## Abbreviations Used

3-MT: 3-methoxytyramine
CDH: cumulative degree hours
FACTS: Forensic Anthropology Center at Texas State
fTMA: free trimethylammonium
LC-MS: liquid chromatography-mass spectrometry
PMI: postmortem interval
